# Comparative speed of kill of sarolaner (Simparica^™^) and afoxolaner (NexGard^®^) against induced infestations of *Ctenocephalides felis* on dogs

**DOI:** 10.1186/s13071-016-1372-1

**Published:** 2016-02-19

**Authors:** Robert H. Six, Julian Liebenberg, Nicole A. Honsberger, Sean P. Mahabir

**Affiliations:** Zoetis, Veterinary Medicine Research and Development, 333 Portage St., Kalamazoo, MI 49007 USA; ClinVet International (pty) Ltd, Uitsigweg, Bainsvlei, 9338 Bloemfontein Republic of South Africa

**Keywords:** *Ctenocephalides felis*, Sarolaner, Simparica™, Afoxolaner, Speed of kill, Flea, Dog, Oral

## Abstract

**Background:**

Fleas are the most common ectoparasite infesting dogs globally. The many possible sequellae of infestation include: direct discomfort; allergic reactions; and the transmission of pathogens. Rapid speed of kill is an important characteristic for a parasiticide in order to alleviate the direct deleterious effects of fleas, reduce the impact of allergic responses, and break the flea infestation cycle. In this study, the speed of kill of a novel orally administered isoxazoline parasiticide, sarolaner (Simparica^™^) against fleas on dogs was evaluated and compared with afoxolaner (NexGard^®^) for 5 weeks after a single oral dose.

**Methods:**

Twenty-four dogs were randomly allocated to treatment with a single oral dose at label rate of either sarolaner (2 to 4 mg/kg) or afoxolaner (2.5 to 6.8 mg/kg) or placebo, based on pretreatment flea counts. Dogs were combed and live fleas counted at 8, 12 and 24 h after treatment and subsequent re-infestations on Days 7, 14, 21, 28 and 35. Efficacy was determined at each time point relative to counts for placebo dogs.

**Results:**

There were no adverse reactions to treatment. A single oral dose of sarolaner provided ≥98.8 % efficacy (based on geometric means) within 8 h of treatment or subsequent weekly re-infestations of fleas to Day 35. By 12 h, fleas were virtually eradicated from all dogs, with only two fleas recovered from a single sarolaner-treated dog on Day 7; efficacy was 100 % at all other time points. Significantly greater numbers of live fleas were recovered from afoxolaner-treated dogs at 8 h on all days and at 12 h on Days 28 and 35 (*P* < 0.05).

**Conclusions:**

In this controlled laboratory evaluation, sarolaner had a significantly faster speed of kill against fleas than afoxolaner. This was noticeably more evident towards the end of the treatment period. The rapid and consistent kill of fleas within 8 to 12 h after a single oral dose of sarolaner over 35 days indicates that this treatment will provide highly effective control of flea infestations, relief for dogs afflicted with flea allergy dermatitis, and should reduce the risk of flea-borne pathogen transmission.

## Background

Flea infestation of pets and the home are a common occurrence and their control and elimination can become an expensive and time consuming challenge [1]. The cat flea, *Ctenocephalides felis felis,* is the most abundant ectoparasite of dogs and cats worldwide [2], and they cause flea allergic dermatitis (FAD), serve as the vector of various bacterial pathogens, and are the intermediate host for filarid and cestode parasites. FAD has been recognized as the most common dermatologic disease of dogs [3]. *Ctenocephalides felis* has been implicated in the transmission of *Rickettsia typhi*, *Rickettsia felis*, *Bartonella henselae*, *Mycoplasma haemofelis* and, in rare cases, *Yersinia pestis* [4–7]. *Ctenocephalides felis* also serves as the intermediate host for the nonpathogenic subcutaneous filarid nematode of dogs, *Acanthocheilonema (Dipetalonema) reconditum*, as well as several species of cestodes including *Dipylidium caninum* and *Hymenoleptis* spp*.* [1, 8, 9].

Effective control of fleas and their associated conditions and diseases is dependent upon the rapid removal of live fleas from the host. Fleas feed almost immediately on attaining a host [10], and direct irritation and allergic reactions are dependent upon the frequency and duration of feeding [11]. Similarly, the longer that a host is exposed to feeding fleas the greater the chance of the transmission of flea-borne pathogens. The use of highly effective, topical and systemic parasiticides has allowed the primary means of flea control to be via direct treatment of the pet. These treatments have largely eliminated the need to treat indoor and outdoor environments, and their use as host targeted therapies markedly reduces the severity and prevalence of FAD [2]. Orally administered compounds, whether administered daily like nitenpyram [12] or monthly like spinosad [13] have been introduced that provide rapid systemic control of fleas for immediate daily treatment, nitenpyram [12], or for up to a month following a single dose, spinosad [13]. These applications have been readily accepted by veterinarians and dog owners for their efficacy, ease of use and the fact that these systemic agents are not topically applied with the resultant lower exposure of the owner or children to potentially toxic residues. Recently, a new class of compounds, the isoxazolines, have shown excellent efficacy against both fleas and ticks for a month or longer following a single orally administered dose [14, 15].

Sarolaner (Simparica^™^, Zoetis) is a new isoxazoline effective against fleas and ticks for at least one month following a single dose. A laboratory study was conducted to compare the speed of kill of a single dose of sarolaner (Simparica^™^, Zoetis) and afoxolaner (NexGard^®^, Merial) against an existing flea (*C. felis*) infestation and subsequent re-infestations for a period of 5 weeks after treatment.

## Methods

### Ethical approval

The study was a masked, negative controlled, randomized, laboratory efficacy design conducted in the Republic of South Africa. Procedures were in accordance with the World Association for the Advancement of Veterinary Parasitology (WAAVP) guidelines for evaluating the efficacy of parasiticides for the treatment, prevention and control of flea and tick infestation on dogs and cats [16] and complied with the principals of Good Clinical Practices [17]. The protocol was reviewed and approved by the local Institutional Animal Care and Use Committee. Masking of the study was assured through the separation of functions. All personnel conducting observations or animal care or performing infestations and counts were masked to treatment allocation.

### Animals

Twenty-four, male and female, purpose-bred mongrel dogs ranging in age from 15 to 105 months and weighing from 7.8 to 21.0 kg were used in the study. All dogs had undergone an adequate wash-out period to ensure that no residual ectoparasiticide efficacy remained from any previously administered compound. Each dog was individually identified by electronic transponder. The dogs were acclimatized to study conditions for a minimum of 14 days before treatment on Day 0. Dogs were individually housed in indoor runs such that no physical contact was possible between dogs. Dogs were fed an appropriate maintenance ration of a commercial dry canine feed, and water was available *ad libitum*. All dogs were given a physical examination to evaluate general health and suitability for inclusion into the study. General health observations were performed twice daily from the start of the acclimation period to the end of the study.

### Design

The study followed a randomized complete block design. Dogs were ranked according to decreasing flea counts into blocks of three and within each block a dog was randomly allocated to treatment with placebo, sarolaner, or afoxolaner. There were eight dogs per treatment group. Dogs were infested with fleas prior to treatment and then weekly following treatment for 5 weeks. Flea counts were conducted at 8, 12 and 24 h after treatment and each subsequent weekly re-infestation.

### Treatment

Day -1 bodyweights were used to determine the appropriate dose to be administered. On Day 0, dogs received either a placebo tablet, a sarolaner chewable tablet (Simparica^™^) to deliver sarolaner at the minimum label dose of 2 mg/kg (range 2 to 4 mg/kg), or NexGard^®^ per label directions (afoxolaner at 2.5 to 6.8 mg/kg). All doses were administered by hand pilling to ensure accurate and complete dosing. Each dog was observed for several minutes after dosing for evidence that the dose was swallowed, and for general health at 1, 4 and 24 h after treatment administration.

### Flea infestation and assessment

The *C. felis* used in the study were from a locally maintained laboratory colony of European origin. The colony was initiated in 2010 with fleas from Germany; fleas obtained from Ireland were introduced into the colony in 2012. Flea infestations were performed on Days -7 (host suitability and allocation), -1, 7, 14, 21, 28 and 35. At each infestation a precounted aliquot of 100 (±5) adult, unfed *C. felis* were directly applied to the animal which was gently restrained for a few minutes to allow the fleas to penetrate and disperse into the hair coat. Each dog was examined and combed to remove and count fleas at 24 h after the initial host suitability infestation, and at 8 (±1), 12 (±1) and 24 (±2) hours after treatment and each subsequent weekly re-infestation. Fleas were replaced on the dogs immediately after each 8 and 12 h evaluation, and discarded after the 24 h counts.

Flea counts were performed by personnel trained in the standard procedures in use at the test facility. Commercial fine-toothed flea combs were used. Dogs were combed using repeated strokes initially while standing starting from the head, then proceeding caudally along the dorsum. The dog was then placed on each side and then on its back for combing of the sides and ventral surfaces. After a few combing strokes were completed, the comb was examined and hair and fleas were removed from the comb and all live fleas were counted. Each animal was examined for a minimum of 10 min; if any fleas were recovered in the last minute, combing was continued in one-minute increments until no fleas were detected.

### Statistical analysis

The individual dog was the experimental unit and the primary endpoint was the live flea count. Data for post-treatment flea counts were summarized with arithmetic and geometric means by treatment group and timepoint. Flea counts were transformed (log_e_(count + 1)) prior to analysis in order to stabilize the variance and normalize the data. Using the PROC MIXED procedure (SAS 9.2, Cary NC), transformed counts were analyzed using a mixed linear model. The fixed effects were treatment, timepoint and the interaction between timepoint and treatment by timepoint. The random effects included room, block within room, block by treatment interaction within room, and error. Testing was two-sided at the significance level α = 0.05.

The assessment of efficacy was based on the percent reduction in the arithmetic and geometric mean live flea counts relative to placebo calculated using Abbott’s formula:

$$ \%\ \mathrm{reduction}=100 \times \frac{\mathrm{mean}\ \mathrm{count}\ \left(\mathrm{placebo}\right)\hbox{--} \mathrm{mean}\ \mathrm{count}\ \left(\mathrm{treated}\right)}{\mathrm{mean}\ \mathrm{count}\ \left(\mathrm{placebo}\right)} $$

## Results

There were no treatment-related adverse events observed during the study. Placebo-treated dogs maintained good flea infestations throughout the study and these counts were maintained even following the combing and re-infestation procedures at 8 and 12 h (Tables 1, 2 and 3).

**Table 1:** Mean live flea counts and efficacy relative to placebo at 8 h after treatment and post treatment re-infestations for dogs treated with a single oral dose of sarolaner or afoxolaner on Day 0

Treatment		Day of treatment or re-infestation					
		0	7	14	21	28	35
Placebo	Range	44–99	51–91	69–89	55–92	62–97	75–100
	A. mean	69.9	74.9	77.0	79.8	79.1	87.5
	G. mean^1^	68.1^a^	73.3^a^	76.6^a^	78.8^a^	78.4^a^	87.1^a^
Sarolaner	Range	0–0	0–3	0–0	0–0	0–8	0–5
	A. mean	0.0	0.4	0.0	0.0	1.6	0.6
	Efficacy (%)	100	99.5	100	100	97.9	99.3
	G. mean^1^	0.0^c^	0.2^c^	0.0^c^	0.0^c^	1.0^c^	0.3^c^
	Efficacy (%)	100	99.7	100	100	98.8	99.7
	*P*-value vs. placebo	<0.0001	<0.0001	<0.0001	<0.0001	<0.0001	<0.0001
Afoxolaner	Range	0–2	0–35	0–31	0–44	3–66	0–71
	A. mean	0.6	6.1	10.1	15.3	27.5	25.0
	Efficacy (%)	99.1	91.8	86.9	80.9	65.2	71.4
	G. mean^1^	0.4^b^	2.4 ^b^	6.8^b^	8.5^b^	18.6^b^	11.3^b^
	Efficacy (%)	99.4	96.7	91.1	89.2	76.3	87.0
	*P*-value vs. placebo	<0.0001	<0.0001	<0.0001	<0.0001	0.0005	0.0005
	*P*-value vs. sarolaner	0.0499	0.0094	<0.0001	<0.0001	<0.0001	<0.0001

^1^Geometric means within columns with the same superscript are not significantly different (*P* > 0.05)

**Table 2:** Mean live flea counts and efficacy relative to placebo at 12 h after treatment and post treatment re-infestations for dogs treated with a single oral dose of sarolaner or afoxolaner on Day 0

Treatment		Day of treatment or re-infestation					
		0	7	14	21	28	35
Placebo	Range	40–95	33–79	60–89	48–88	56–85	62–95
	A. mean	62.3	59.5	74.6	75.8	70.0	80.4
	G. mean^1^	60.4^a^	57.0^a^	74.1^a^	74.7^a^	69.3^a^	79.8^a^
Sarolaner	Range	0–0	0–2	0–0	0–0	0–0	0–0
	A. mean	0.0	0.3	0.0	0.0	0.0	0.0
	Efficacy (%)	100	99.6	100	100	100	100
	G. mean^1^	0.0^b^	0.1^b^	0.0^b^	0.0^b^	0.0^c^	0.0^c^
	Efficacy (%)	100	99.7	100	100	100	100
	*P*-value vs.placebo	<0.0001	<0.0001	<0.0001	<0.0001	<0.0001	<0.0001
Afoxolaner	Range	0–0	0–2	0–2	0–4	0–28	0–25
	A. mean	0.0	0.3	0.3	0.9	5.5	4.3
	Efficacy (%)	100	99.6	99.7	98.8	92.1	94.7
	G. mean^1^	0.0^b^	0.1^b^	0.1^b^	0.5^b^	2.5^b^	1.4^b^
	Efficacy (%)	100	99.7	99.8	99.4	96.4	98.2
	*P*-value vs. placebo	<0.0001	<0.0001	<0.0001	<0.0001	<0.0001	<0.0001
	*P*-value vs. sarolaner	1.000	1.000	0.2455	0.0713	0.0008	0.0161

^1^Geometric means within columns with the same superscript are not significantly different (*P* > 0.05)

**Table 3:** Mean live flea counts and efficacy relative to placebo at 24 h after treatment and post treatment re-infestations for dogs treated with a single oral dose of sarolaner or afoxolaner on Day 0

Treatment		Day of treatment or re-infestation					
		0	7	14	21	28	35
Placebo	Range	36–87	28–88	48–87	53–73	43–73	58–83
	A. mean	56.5	59.3	64.3	66.3	59.8	71.4
	G. mean^1^	54.6^a^	56.6^a^	62.7^a^	66.0^a^	59.0^a^	70.8^a^
Sarolaner	Range	0–0	0–1	0–0	0–0	0–0	0–0
	A. mean	0.0	0.1	0.0	0.0	0.0	0.0
	Efficacy (%)	100	99.8	100	100	100	100
	G. mean^1^	0.0^b^	0.1^b^	0.0^b^	0.0^b^	0.0^b^	0.0^b^
	Efficacy (%)	100	99.8	100	100	100	100
	*P*-value vs.placebo	<0.0001	<0.0001	<0.0001	<0.0001	<0.0001	<0.0001
Afoxolaner	Range	0–0	0–0	0–0	0–0	0–0	0–0
	A. mean	0.0	0.0	0.0	0.0	0.0	0.0
	Efficacy (%)	100	100	100	100	100	100
	G. mean^1^	0.0^b^	0.0^b^	0.0^b^	0.0^b^	0.0^b^	0.0^b^
	Efficacy (%)	100	100	100	100	100	100
	*P*-value vs. placebo	<0.0001	<0.0001	<0.0001	<0.0001	<0.0001	<0.0001
	*P*-value vs. sarolaner	1.000	0.5043	1.000	1.000	1.000	1.000

^1^Geometric means within columns with the same superscript are not significantly different (*P* > 0.05)

**Fig. 1:**
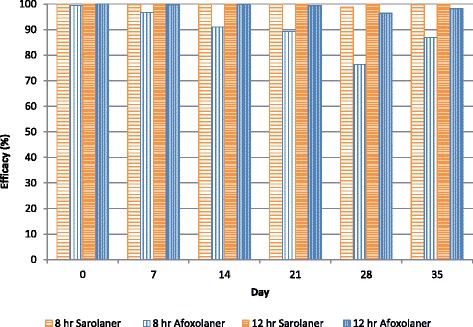
Percent efficacy based on geometric mean counts relative to placebo at 8 and 12 h after treatment and weekly post treatment re-infestations of fleas for dogs treated with a single oral dose of sarolaner or afoxolaner on Day 0

At the 8-h time point, both treatments resulted in significantly lower flea counts than placebo-treated dogs (*P* ≤ 0.0005) throughout the study (Table 1). Treatment with sarolaner also resulted in significantly lower flea counts than afoxolaner at 8 h on all assessment days (*P* ≤ 0.0499) and the sarolaner treatment provided greater and more consistent efficacy at 8 h (≥98.8 %, ≥97.9 % geometric, arithmetic mean) for the entire study. Efficacy based on geometric means for afoxolaner ranged from 76.3 to 99.4 %, and on arithmetic means from 65.2 to 99.1 % (Table 1). Notably, the efficacy for the afoxolaner-treated dogs declined as the study progressed, and was <90 % from Day 21 onwards (Fig. 1).

At the 12-h timepoint, both treatments resulted in significantly lower flea counts than placebo-treated dogs (*P* ≤ 0.0001) throughout the study (Table 2) and flea counts were significantly higher for afoxolaner-treated dogs on Days 28 and 35 (*P* ≤ 0.0161). Efficacy was very high for sarolaner with only two live fleas found on one dog on Day 7 (efficacy of 99.6 %); on all other days efficacy for sarolaner was 100 %. For afoxolaner, 100 % efficacy was only achieved on Day 0, and efficacy tended to decline as the study progressed (Fig. 1). On Days 28 and 35, efficacy for afoxolaner-treated dogs was <95 % (arithmetic mean) (Table 2).

At the 24-h time point, both treatments resulted in significantly lower flea counts than placebo-treated dogs (*P* ≤ 0.0001) throughout the study (Table 3). Both products provided ≥99.8 % reduction in mean live flea counts through Day 35 with 100 % efficacy at most evaluations (Table 3); there was no difference between the counts for the two products on any assessment day (*P* ≥ 0.5043).

## Discussion

For any parasiticide product providing flea control, the product’s initial speed of kill is important to provide the pet with rapid relief from the existing infestation. The residual speed of kill is critical to decrease the likelihood of transmission of vector borne disease, ensure that any newly acquired fleas are rapidly eliminated before they can reproduce, and assist in the management of flea allergic dermatitis. The added benefit for products prescribed by a veterinarian is instilling pet owner confidence in the outcome of the treatment and the treatment recommendation which can enhance compliance. A single oral dose of sarolaner at the proposed commercial dose of 2 to 4 mg/kg resulted in the rapid reduction of an existing flea infestation as well as rapid kill of newly infested fleas for 35 days. Treatment with sarolaner resulted in effective control of greater than 97 % within 8 h after treatment and weekly re-infestations for 35 days, and fleas were virtually eliminated from all dogs within 12 h over the entire 35 days. This rapid efficacy will disrupt the flea life cycle by killing adult fleas before they can lay eggs, thus reducing the environmental infestation level [18, 19].

The efficacy provided by sarolaner at 8 h was significantly greater than that provided by afoxolaner at all evaluations but especially when performance is compared towards the end of the monthly treatment period when the efficacy of afoxolaner declined below 90 % from Day 21 onwards. Efficacy of afoxolaner was improved at 12 h and again sarolaner had consistently better kill of fleas and there were significantly more live fleas recovered from the afoxolaner-treated dogs on Days 28 and 35; sarolaner was 100 % effective on these days. There was no difference between sarolaner and afoxolaner at the 24 h assessments. The results for afoxolaner in this study are in agreement with those reported by Hunter et al. [20] who reported 99.6 % efficacy at 12 h and 100 % efficacy at 24 h against an existing infestation and efficacy ranging from 89.7 to 99.5 % at 12 h and ≥99.9 % efficacy at 24 h for 35 days.

The rapid and consistent speed of kill of fleas over a period of 35 days makes sarolaner an excellent option for monthly flea control that will reduce the direct irritation caused by flea infestation, assist in the prevention of FAD, and should reduce the risk of flea-borne diseases.

## Conclusions

A single oral dose of sarolaner at 2 to 4 mg/kg resulted in more rapid control of an existing flea infestation than afoxolaner with >97 % efficacy within 8 h of both treatment and subsequent re-infestations for 35 days. Fleas were virtually eliminated from all dogs within 12 h. Efficacy of sarolaner at 8 h was significantly superior to afoxolaner on all days, and at 12 h on Days 28 and 35. Afoxolaner’s efficacy at 12 h decreased towards the end of the month long treatment interval. The rapid and consistent speed of kill provided by sarolaner over the 5 weeks following a single dose can provide peace of mind for veterinarians and pet owners that there will be no gap in protection against fleas when sarolaner is dosed monthly, will reduce the deleterious effects of flea infestation and the clinical signs of flea allergy dermatitis, and should reduce the risk of flea-borne pathogen transmission.

## References

[1] Blagburn BL, Dryden MW (2009). Biology, treatment and control of flea and tick infestations. Vet Clin Small Anim.

[2] Rust MK (2005). Advances in the control of *Ctenocephalides felis felis* (cat flea) on cats and dogs. Trends Parasitol.

[3] Scott DW, Miller WH, Griffin CE (2001). Muller and Kirk’s small animal dermatology.

[4] Breitschwerdt EB (2008). Feline bartonellosis and cat scratch disease. Vet Immunol Immunopathol.

[5] Kamrani A, Parreira VR, Greenwood J, Prescott J (2008). The prevalence of Bartonella, hemoplasma, and Rickettsia felis infections in domestic cats and in cat fleas in Ontario. Can J Vet Res.

[6] Brewer MM, Woods JE, Hawley JR, Wisnewski N, Lappin M (2005). Evaluation of experimental transmission of Candidatus Mycoplasma haemominutum and Mycoplasma haemofelis by Ctenocephalides felis to cats. Am J Vet Res.

[7] Eisen RJ, Borchert JN, Holmes JL, Amatre G, Van Wyk K, Enscore RE (2008). Early-phase transmission of *Yersinia pestis* by cat fleas (*Ctenocephalides felis*) and their potential role as vectors in a plague-endemic region of Uganda. Am J Trop Med Hyg.

[8] Pugh RE (1987). Effects on the development of *Dipylidium caninum* and on the host reaction to this parasite in the adult flea (*Ctenocephatides felis felis*). Parasitol Res.

[9] Marshall AG (1967). The cat flea, *Ctenocephalides felis felis* (Bouche, 1835) as an intermediate host for cestodes. Parasitology.

[10] Dryden MW, Gaafar SM (1991). Blood consumption by the cat flea, *Ctenocephalides felis* (Siphonaptera: Pulicidae). J Med Entomol.

[11] Dryden MW (2009). Flea and tick control in the 21st century: challenges and opportunities. Vet Dermatol.

[12] Rust MK, Waggoner MM, Hinkle NC, Stansfield D, Barnett S (2003). Efficacy and longevity of nitenpyram against adult cat fleas (Siphonaptera: Pulicidae). J Med Entomol.

[13] Snyder DE, Meyer J, Zimmermann AG, Qiao M, Gissendanner SJ, Cruthers LR (2007). Preliminary studies on the effectiveness of the novel pulicide, spinosad, for the treatment and control of fleas on dogs. Vet Parasitol.

[14] Rohdich N, Roepke RKA, Zschiesche E (2014). A randomized, blinded, controlled and multi-centered field study comparing the efficacy and safety of Bravecto^®^ (fluralaner) against Frontline^®^ (fipronil) in flea- and tick-infested dogs. Parasitol Vectors.

[15] Shoop WL, Harline EJ, Gould BR, Waddell ME, McDowell RG, Kinney JB (2014). Discovery and mode of action of afoxolaner, a newisoxazoline parasiticide for dogs. Vet Parasitol.

[16] Marchiondo AA, Holdsworth PA, Fourie LJ, Rugg D, Hellmann K, Snyder DE (2013). World Association for the Advancement of Veterinary Parasitology (W.A.A.V.P.) 2nd. ed.: Guidelines for evaluating the efficacy of parasiticides for the treatment, prevention and control of flea and tick infestations on dogs and cats. Vet Parasitol.

[17] EMEA. Guideline on good clinical practices. VICH Topic GL9. http://www.ema.europa.eu/docs/en_GB/document_library/Scientific_guideline/2009/10/WC500004343.pdf. Accessed 23 Aug 2015.

[18] Jacobs DE, Hutchinson MJ, Ryan WG (2001). Control of flea populations in a simulated home environment model using lufenuron, imidacloprid or fipronil. Med Vet Entomol.

[19] Dryden MW, Payne PA, Lowe A, Mailen S, Smith V, Rugg D (2007). Efficacy of a topically applied formulation of metaflumizone on cats against the adult cat flea, flea egg production and hatch, and adult flea emergence. Vet Parasitol.

[20] Hunter JS, Dumont P, Chester TS, Young DR, Fourie JJ, Larsen DL (2014). Evaluation of the curative and preventive efficacy of a single oral administration of afoxolaner against cat flea *Ctenocephalides felis* infestations on dogs. Vet Parasitol.

